# Severe Visual Impairment and Blindness in Infants: Causes and Opportunities for Control

**DOI:** 10.4103/0974-9233.80698

**Published:** 2011

**Authors:** Parikshit Gogate, Clare Gilbert, Andrea Zin

**Affiliations:** 1 Dr. Gogate’s Eye Clinic, Pune, India; 2 Lions NAB Eye Hospital, Miraj, India; 3 International Centre for Eye Health, London school of Hygiene and Tropical Medicine, London, UK, India; 4 Instituto Fernandes Figueira, FIOCRUZ, Rio de Janeiro, Brazil, India

**Keywords:** Childhood Blindness, Congenital Anomalies, Congenital Cataract, Retinopathy of Prematurity

## Abstract

Childhood blindness has an adverse effect on growth, development, social, and economic opportunities. Severe visual impairment (SVI) and blindness in infants must be detected as early as possible to initiate immediate treatment to prevent deep amblyopia. Although difficult, measurement of visual acuity of an infant is possible. The causes of SVI and blindness may be prenatal, perinatal, and postnatal. Congenital anomalies such as anophthalmos, microphthalmos, coloboma, congenital cataract, infantile glaucoma, and neuro-ophthalmic lesions are causes of impairment present at birth. Ophthalmia neonatorum, retinopathy of prematurity, and cortical visual impairment are acquired during the perinatal period. Leukocoria or white pupillary reflex can be cause by congenital cataract, persistent hyperplastic primary vitreous, or retinoblastoma. While few medical or surgical options are available for congenital anomalies or neuro-ophthalmic disorders, many affected infants can still benefit from low vision aids and rehabilitation. Ideally, surgery for congenital cataracts should occur within the first 4 months of life. Anterior vitrectomy and primary posterior capsulotomy are required, followed by aphakic glasses with secondary intraocular lens implantation at a later date. The treatment of infantile glaucoma is surgery followed by anti-glaucoma medication. Retinopathy of prematurity is a proliferation of the retinal vasculature in response to relative hypoxia in a premature infant. Screening in the first few weeks of life can prevent blindness. Retinoblastoma can be debulked with chemotherapy; however, enucleation may still be required. Neonatologists, pediatricians, traditional birth attendants, nurses, and ophthalmologists should be sensitive to a parent’s complaints of poor vision in an infant and ensure adequate follow-up to determine the cause. If required, evaluation under anesthesia should be performed, which includes funduscopy, refraction, corneal diameter measurement, and measurement of intraocular pressure.

## INTRODUCTION

Visual impairment and blindness in children pose a special problem for ophthalmologists, as many eye care practitioners are not familiar with performing pediatric eye examinations and measuring visual acuity in infants. Infants are unable to verbalize their complaints, and history from parents and care takers may lack important details. The first year of life is also the time when the visual system develops and binocular vision is formed.^1^ If a visual deficit at this age is not treated in a timely manner, amblyopia and permanent visual deficit can occur. Hence, early diagnosis and prompt treatment is essential. The burden of blindness measured in blind-person years due to childhood blindness is second only to cataract – the most common cause of avoidable blindness in childhood.^2^ Studies worldwide show that many of the causes of blindness in children are either preventable or treatable (ie, avoidable).^3^ Even children who have visual loss that cannot be clinically treated, can be helped with low vision devices and rehabilitation. Childhood blindness affects the individual, their family, and the community. Blindness also has implications for infants’ development, education, and future social, marital, and economic prospects. Nearly 75% of early learning comes from vision. Early onset visual loss can have profound consequences on a child’s motor, social, emotional, and psychological development.^4^

The World Health Organization (WHO) classifies the causes of childhood blindness according to the anatomical site most affected and the underlying etiology.^5^ Visual loss in infants can be either prenatal (ie, occurring at the time of conception or during the intrauterine period) or postnatal (during or after birth). Prenatal causes are congenital anomalies – anophthalmos, microphthalmos, and coloboma; congenital cataract, retinal dystrophies such as Leber’s congenital amaurosis, infantile glaucoma, and congenital cloudy cornea. In the perinatal period (ie, from the 28^th^ week of gestation through to 1-4 weeks after birth), the following conditions can occur: cortical impairment from birth asphyxia, ophthalmia neonatorum, and retinopathy of prematurity. Postnatal conditions (ie, those acquired after birth) are unusual during infancy. As retinoblastoma can be inherited as an autosomal dominant condition, it is considered a prenatal condition for the purposes of this paper.

Leukocoria may be seen in early infancy can be due to congenital cataract, retinopathy of prematurity (ROP), persistent hyperplastic primary vitreous (PHPV) – anterior or posterior – retinoblastoma or rare genetic disorders such as Norrie’s disease. A leukocoria detected by parents in later infancy may be due to developmental cataract or retinoblastoma. Retinoblastoma is the most common childhood tumor presenting at the age of 12-15 months in bilateral cases and 18-24 months in unilateral cases; it can also develop much later in childhood. It is essential to examine children presenting with leukocoria under anesthesia due to the potentially serious underlying causes.

## PRENATAL ONSET

### Congenital anomalies

Anophthalmos and microphthalmos are congenital anomalies in which the entire eyeball is entirely absent or smaller than normal from birth. Typical complete uveal coloboma is the most common congenital anomaly in which the embryonic fissure does not close completely by the sixth week of intrauterine life. Coloboma can cause severe visual loss if the defect involves the macula and/or optic nerve. Surveys from schools for the blind in many parts of Asia show that these congenital eye anomalies are a leading cause of blindness and severe visual impairment in children.^6^–^9^ These are difficult to research, even in countries with the best healthcare systems and expertise. In the majority of cases, even with in-depth molecular genetic testing and detailed investigation, no cause can be identified.^10^ This is because these anomalies are likely to be due to genes controlling eye development (which are largely unknown) and possibly, gene-environment interactions, reflecting similar processes elucidated for folic acid and spina bifida.^11^ Even if an environmental agent (eg, folate) could be identified, the intervention would have to been extremely broad to have a measurable effect, be inexpensive and safe (as it would need to be given to large numbers of people) and be nonteratogenic – a difficult proposition in developing countries.

Medical therapy or surgery are rarely effective in congenital cases; however, refraction and low vision aids can help many of these cases.^12^ Many children with congenital defects can be taught to read print with low vision aids instead of Braille.

PHPV is a congenital anomaly in which the primary vitreous, which extends from the optic disc to the vitreous base at the posterior pole of the lens, persists into childhood.^13^ This anomaly can cause severe visual loss, and may be associated with microphthalmos. PHPV is usually unilateral, although bilateral cases can occur. Early vitrectomy can be attempted in eyes where the surrounding vitreous is clear and the retina fairly developed; however, the visual outcomes are often suboptimal.^14^

### Infantile glaucoma

Infantile glaucoma can result in blindness without early intervention.^15^ Surgery followed by medication and optical aids is the treatment of choice. Numerous genes have been implicated and genetic counselling is essential if more than one sibling is affected.

### Retinal dystrophies

Retinal dystrophies have variable genetic inheritance and are common in communities with high rates of consanguinity. Although the dystrophy may only give visual symptoms later in life, this is a common cause of blindness in children worldwide, particularly in Middle Eastern countries.^16^,^17^ Low vision aids are effective for navigation and reading for individuals affected with retinal dystrophies. Genetic counselling is required if more than one sibling is involved.

Leber’s congenital amaurosis is an autosomal recessive condition that manifests in infancy. The fundus may be normal or show peripheral chorioretinal atrophy and granularity with nystagmus. Oculodigital syndrome, in which the infant constantly rubs or presses on the eyes, which leads to enophthalmos, is common. Other common features include learning disability, deafness, and epilepsy. In Knobloch syndrome nystagmus, high myopia and late retinal detachment are seen. Oculocutaneous albinism, which may be X-linked or autosomal recessive, is detectable at birth. Ocular albinism affects only male infants due to the X-linked inheritance and is usually diagnosed at infancy. Achromatopsy is a rare autosomal recessive heterogeneous group of stationary retinal dystrophies characterized by reduced central vision, poor color vision, and normal fundus. The usual presentation is reduced vision, marked photophobia, and nystagmus in infancy. A macular cherry red spot could be due to Tay-Sachs disease, which is fatal by the second year of life or due to Niemann-Pick disease or Sandhoff disease.

### Congenital cataract

The birth prevalence of cataract is 3-5/1,000 live births.^18^ While monogenetic abnormalities, syndromes, metabolic disorders, genetic disorders, and congenital rubella syndrome are recognized causes of this condition, in the majority, the cause is unknown.^19^ The importance of congenital cataract is increasing as a cause of blindness among children in developing countries, with other causes declining. There can be 2 years delay from cataract diagnosis to presentation for surgery.^20^ Early recognition and referral is essential to prevent development of dense amblyopia. However, the timing of intraocular lens (IOL) implantation remains controversial.^21^ Many experienced surgeons are implanting IOLs in very young children, ie, 12 months or older. While children with unilateral cataract have undergone IOL implantation at as early as 6 months of age, it is better to wait until 2 years of age for implanting IOLs in children with bilateral cataracts.^21^,^22^ In \ children where the risk of general anesthesia very high, bilateral surgery at the same visit is advisable; however, this should be an exception rather than the rule. There is almost a hundred percent posterior capsular opacification in pediatric eyes. Experts recommend primary posterior capsulotomy and anterior vitrectomy in children up to the age of 6-7 years. At 6 years or older, the child can sit on a slit lamp for Nd:YAG laser capsulotomy after surgery, if required.^21^ The anterior vitrectomy removes the almost solid vitreous base, which can act as a scaffold for migrating lenticular cells. The pars plana approach has the advantage of keeping the primary incision and the anterior chamber free of vitreous, but this is technically demanding for anterior segment surgeons. Moreover, there is an exaggerated fear of suprachoroidal hemorrhage.^23^ Vitrectomy through limbal side ports is easier but the surgeon has to be meticulous in removing vitreous from the anterior chamber. The primary posterior capsulotomy can be performed before or after inserting the intraocular lens. There is a risk of the capsulotomy extending during the implantation of a foldable lens. An MVR blade may be used to make a ’nick’ below the implanted lens to later enlarge the opening with a vitrectors.^23^ A-scan biometry and keratometry need to be performed under anesthesia. Hydrophobic acrylic or poly-methyl-methacrylate IOLs are best for infants. Generally, the target postoperative refraction is emmetropia by the age of 5-6 years. Aphakic glasses have to be corrected for near vision.

Pediatric cataract surgery is one step in a series of interventions required to rehabilitate vision.^24^ Postoperative management includes frequent steroid eye drops with cycloplegia, followed by early, accurate, and repeated refractions and treatment of amblyopia by patching the better seeing eye. Late complications of pediatric IOL implantation include opacification of the visual axis and glaucoma, the latter being particularly difficult to treat. Parents must be counselled about the need for regular ophthalmic examination, refraction, and intraocular pressure measurement. It is important to remember that aphakic/pseudophakic children need correction for near as well as distance, and many are able to tolerate executive bifocals very well.^21^,^24^

### Retinoblastoma

Retinoblastoma is the most common intraocular malignancy in early childhood. It usually presents after infancy as leukocoria, esotropia, or masquerades as uveitis. Lesions detected early can be treated with chemotherapy with preservation of the globe; however, larger lesions may need enucleation.^25^,^26^ A prosthesis needs to be implanted in the child’s eye to prevent the contracture of the socket. A mule’s implant wrapped in a sclera shell or dermal fat grafts may be used for this purpose. Management with specialist tertiary oncology services is also advisable.

## PERINATAL ONSET

### Ophthalmia neonatorum

This is an eminently preventable condition in which the eyes of the infant become infected in the birth passage during delivery. Credé’s prophylaxis and antenatal testing or treatment of sexually transmitted diseases during pregnancy can prevent the condition. Credé’s prophylaxis entails cleaning the infants’ eyes immediately after birth with instillation of a topical antibiotic (eg, tetracycline eye ointment) or antiseptic (eg, 2.5% povidone iodine). Traditionally, a 2% solution of silver nitrate was used for Credé’s prophylaxis. The agent used depends on the local epidemiology of sexually transmitted diseases and the organism’s sensitivity to antibiotics. Treatment of ophthalmia neonatorum must entail systemic as well as topical treatment, to ensure treatment of extraocular sites of infection (eg, pneumonia).

### Retinopathy of prematurity

Retinopathy of prematurity (ROP) is responsible for up to 15% of all causes of blindness in developed countries and up to 60% in middle income countries.^27^ A number of risk factors are implicated in the development of ROP. However, the most commonly identified risk factors are the degree of immaturity measured by birth weight (BW), gestational age (GA), and prolonged exposure to supplementary oxygen.^28^ Paradoxically, the hyperbaric oxygen that saves an infant’s life, if not properly controlled and tapered, can cause blindness. There are several trials seeking to define safe upper and lower limits of arterial oxygen saturation.^29^ The improvement in neonatal care can lead to an increase in survival rates of premature infants in middle-income countries and major metropolises of even poorer countries. As a consequence, ROP has become a very important cause of childhood in these developing economies, where quality of neonatal care still needs to improve. The disease is classified by severity (stages I-V), by site (zones 1-3), and by extent (clock hours 1-12). While most developed countries screen infants by BW < 1,500 g,^30^ middle income and developing countries need larger BW criteria as infants weighing up to 2,500 g are developing severe ROP.^31^,^32^ Appropriate screening protocols ensure early detection in the first weeks of life so that treatment (peripheral ablation of avascular retina) with laser or cryotherapy can be provided. So far, there is no evidence provided by clinical trials that surgery is beneficial for cases in stages IV and V. Anti-vascular endothelial growth factors (VEGFs) are promising drugs for treatment of retinal proliferative diseases in adults. However, extreme caution is warranted in infants, as these drugs interfere with the normal development of vasculature elsewhere in the body and may impede development of the lungs and alveoli of the lungs, which are VEGF-sensitive and develop at the same time as does ROP.^33^ Premature infants are at increased risk of developing refractive errors, strabismus, amblyopia, and low vision; hence, long-term follow-up is recommended.

### Optic nerve lesions and cerebral visual impairment

Optic nerve lesions and cerebral visual impairment are the most common causes of visual impairment in many developed countries^34^ where these are often a consequence of preterm birth.^35^ Birth asphyxia, which causes cerebral palsy, may affect the optic nerve and cause cortical visual impairment. Little can be done by way of medical treatment. Low vision aids and rehabilitation are often the only recourse. Often, such children are severely handicapped, which makes assessment and management even more challenging.

## POSTNATAL ONSET

Keratomalacia cause by acute deficiency of vitamin A is very unusual in the first year of life, and most infants before the age of 9 months (when they are immunized) will have protective measles antibodies received from their mother. The common causes of blindness in childhood such as vitamin A deficiency, measles, trauma, and trachoma are uncommon in infancy. Unless the refractive error is very large [>3-4 diopter (D)], or is causing strabismus, spectacles are not required in infants. However, if the infant has low vision, even small refractive errors may need correction.

## EVALUATION OF A VISUALLY IMPAIRED INFANT

While evaluation of a visually impaired infant may seem challenging for the general ophthalmologist, the task is not that difficult. Infants are best examined in the position they are most comfortable – on their parents’ shoulders, in their laps, or cribs. We recommend not making the infant and parents wait in a busy waiting area. A separate area for breastfeeding is welcome, as this puts the mother and child at ease. The infant can be examined for fixating and following of the flashlight. The ophthalmologist should also have a quick look at the cornea, anterior chamber, lens, and pupillary reflex. A handheld slit lamp is the best instrument for this component of the evaluation. The red reflex should be examined to rule out congenital cataract, advanced ROP, and other causes of leukocoria. If the child is uncooperative or distressed, the examination can be repeated after a few hours or next day after instilling atropine eye ointment to dilate the pupil. However, the drug may affect the pupil for a week. Cyclopentolate 0.1%, tropicamide 0.1%, and phenylephrine 2.5% eye drops may also be instilled, though there is a risk of toxicity due to systemic absorption. Cyclopentolate and tropicamide are parasympatholytic agents acting on sphincter pupillae, whereas phenylephrine is sympathomimetic acting on the dilator papillae muscle. The child can also be given 2.5 ml of phenergan syrup (properazine – a mild sedative and antihistamine combination) and then breastfed to lull it into sleep. This allows more detailed fundus and external ocular evaluation, and measurement of intraocular pressure by non-contact or Perkins (more accurate) tonometer. If a serious lesion such as retinoblastoma is suspected, an evaluation under anesthesia is essential for detailed examination of the fundus. The opportunity must be utilized not just to examine the fundus but to also perform tonometry, refraction, measurement of corneal diameter, and axial length.

Orthoptic evaluation of an infant requires patience. Jampolsky’s dictum of “one toy-one look” should be used to examine the infant’s ocular motility in the nine cardinal directions of gaze. An assortment of soft toys, brightly colored objects, and even mobile phones can be used to arouse the child’s interest. The objects should not make noise as the child will be attracted through the auditory and not the visual signals, which would defeat the purpose of the examination. Visual acuity can be formally assessed using forced preferential looking tests (eg, Cardiff cards), with the child sitting comfortably on a parent’s lap. The test is based on the psychological percept that humans are attracted to novel stimuli. If the child is shown a line drawing at one end of the card sheet and the other end is kept blank, the child will divert its eyes to the drawing rather than the blank area. The line drawings are made progressively finer to estimate higher orders of form vision. Worth’s ivory fall test and small sweets commonly used to decorate cakes, “hundreds and thousands”, can also be used. The child picks up the small sweets if it is able to see them. At first, bilateral vision is tested followed by monocular testing. The mother should be asked to occlude the infant’s eyes one at a time, and report on the infant’s response. If the infant objects to occlusion of one eye, this may indicate the visual acuity of the other eye is poor.

## WHY IS EARLY DETECTION IMPORTANT?

There is a narrow window of opportunity in treating a visually impaired infant. Binocular single vision develops by 6 months of life and a visual deficit, if not detected and treated in time, may leave the child bereft of stereopsis. The amblyopia that develops from visual deprivation of early onset, irrespective of the cause, can be dense and difficult to treat.

Pediatricians, general practitioners, and midwives should be educated and encouraged to perform the red reflex test. Using the direct ophthalmoscope, they should be taught to detect any opacity seen in the infant’s red reflex. All healthcare personnel working for the care of the infant should be sensitized to the eye conditions in infancy and on the causes of childhood blindness and visual impairment. Their training curricula should emphasize on the importance of early detection and treatment of such children. Training of midwives, traditional birth attendants, healthcare workers working for child health and immunization would be of immense help in early detection of such children.

## BARRIERS TO ACCESS OF EYE CARE SERVICE

The major barrier to accessing eye care services for infants is the absence of trained personnel who can diagnose a problem early. Developed countries have established referral systems between family practitioners, health visitors, neonatal units, and pediatric ophthalmology and retina specialist. However, pediatric eye care centers are rare in developing countries. There are financial and geographic barriers for many parents from poorer communities. Diagnostic, curative, and rehabilitative services may not be available in the region. Pediatric eye care interventions are also more expensive than adult treatment. For example, general anesthesia is often required for examination and treatment. This entails liaison with other medical practitioners such as pediatricians, anesthetists, and neonatologists. Another significant barrier is the lack of knowledge on the part of healthcare providers. Many healthcare workers, including physicians ask parents to wait until the child is older before any treatment can be given. Some physicians are unsure about what needs to be done. Others believe that the problem may resolve, as the child grows older or that a unilateral condition does not matter as long as the other eye is healthy. Due to these misconceptions, appropriate referral may too late if dense amblyopia has occurred.

Even when the infant has been referred for treatment appropriately, many parents believe that their infant is too small to undergo surgery or wear spectacles and, in some communities, visual loss in infants is not considered a priority for the family, especially for females.

However, a child who cannot be helped by medicine or surgery may still benefit from use of spectacles and/or low vision aids. Completely and irreversibly blind children can benefit by rehabilitation and special school education. This should be emphasized during parent counselling. Ophthalmologists should be nonjudgmental and empathetic while breaking the news about a potentially blinding condition. Ocular conditions, which have a genetic or familial basis, may be blamed on one of the parents. The ophthalmologist should spend some time with the parents to reassure, counsel, and suggest various alternatives that are available for the infant. It may take the parents some time to come to terms with their child’s visual loss. Frequent ophthalmic visits to confirm the level of vision and make a definitive diagnosis can be difficult for parents. A social worker or counsellor can establish a rapport with the parents or caretakers to ensure that they comply with the treatment as much as possible.

Blindness and severe visual impairment in infants is not that difficult to detect and diagnose. With proper care, most of these infants can be helped and formation of dense amblyopia prevented. Even if the ophthalmologist may not be able to help medically or surgically, optical aids and rehabilitation can help children reach their full capacity.
